# Targeting Treatment-Resistant Auditory Verbal Hallucinations in Schizophrenia with fMRI-Based Neurofeedback – Exploring Different Cases of Schizophrenia

**DOI:** 10.3389/fpsyt.2016.00037

**Published:** 2016-03-15

**Authors:** Miriam S. Dyck, Krystyna A. Mathiak, Susanne Bergert, Pegah Sarkheil, Yury Koush, Eliza M. Alawi, Mikhail Zvyagintsev, Arnim J. Gaebler, Sukhi S. Shergill, Klaus Mathiak

**Affiliations:** ^1^Department of Psychiatry, Psychotherapy and Psychosomatics, Jülich-Aachen Research Alliance (JARA)-Brain, RWTH Aachen University, Aachen, Germany; ^2^Jülich-Aachen Research Alliance (JARA)-Translational Brain Medicine, Jülich, Aachen, Germany; ^3^Department of Child and Adolescent Psychiatry, Psychotherapy and Psychosomatics, RWTH Aachen University, Aachen, Germany; ^4^Institute of Psychiatry, Psychology and Neuroscience, King’s College London, London, UK; ^5^Institute of Bioengineering, Ecole Polytechnique Fédérale de Lausanne (EPFL), Lausanne, Switzerland; ^6^Department of Radiology and Medical Informatics, University of Geneva, Geneva, Switzerland

**Keywords:** schizophrenia, auditory hallucinations, functional magnetic resonance imaging, neurofeedback, brain–computer interface, self-regulation, anterior cingulate cortex, affect

## Abstract

Auditory verbal hallucinations (AVHs) are a hallmark of schizophrenia and can significantly impair patients’ emotional, social, and occupational functioning. Despite progress in psychopharmacology, over 25% of schizophrenia patients suffer from treatment-resistant hallucinations. In the search for alternative treatment methods, neurofeedback (NF) emerges as a promising therapy tool. NF based on real-time functional magnetic resonance imaging (rt-fMRI) allows voluntarily change of the activity in a selected brain region – even in patients with schizophrenia. This study explored effects of NF on ongoing AVHs. The selected participants were trained in the self-regulation of activity in the anterior cingulate cortex (ACC), a key monitoring region involved in generation and intensity modulation of AVHs. Using rt-fMRI, three right-handed patients, suffering from schizophrenia and ongoing, treatment-resistant AVHs, learned control over ACC activity on three separate days. The effect of NF training on hallucinations’ severity was assessed with the Auditory Vocal Hallucination Rating Scale (AVHRS) and on the affective state – with the Positive and Negative Affect Schedule (PANAS). All patients yielded significant upregulation of the ACC and reported subjective improvement in some aspects of AVHs (AVHRS) such as disturbance and suffering from the voices. In general, mood (PANAS) improved during NF training, though two patients reported worse mood after NF on the third day. ACC and reward system activity during NF learning and specific effects on mood and symptoms varied across the participants. None of them profited from the last training set in the prolonged three-session training. Moreover, individual differences emerged in brain networks activated with NF and in symptom changes, which were related to the patients’ symptomatology and disease history. NF based on rt-fMRI seems a promising tool in therapy of AVHs. The patients, who suffered from continuous hallucinations for years, experienced symptom changes that may be attributed to the NF training. In order to assess the effectiveness of NF as a therapeutic method, this effect has to be studied systematically in larger groups; further, long-term effects need to be assessed. Particularly in schizophrenia, future NF studies should take into account the individual differences in reward processing, fatigue, and motivation to develop individualized training protocols.

## Introduction

Auditory verbal hallucinations (AVHs) are a hallmark of schizophrenia and affect approximately 60–80% of patients with schizophrenia (1). They encompass a range of experiences: single or multiple voices; familiar to the patient or unknown; speaking sequentially or simultaneously; speaking in the first, second, or third person; giving commands, comments, insults, or encouragement (2). AVHs constitute a significant persisting burden for the patients (3) and are typically associated with social and occupational dysfunction (4) as well as worse prognosis (5). A large meta-analysis (6) revealed that only 14% of individuals with schizophrenia fully recover over 10 years. The rest will suffer from negative and positive symptoms as well as cognitive deficits of varying intensity.

Although antipsychotic medication rapidly reduces the frequency and severity of hallucinations in the majority of patients (7), AVHs are refractory to traditional antipsychotic drugs in 25–30% of cases (4). Psychosocial treatments, including cognitive behavioral therapy, are often applied as an augmentation to pharmacological treatment, helping to reduce the emotional distress associated with AVHs and aiming at developing new coping strategies (7). However, they rarely change the frequency of hallucinations and moreover, many patients do not manage to engage in therapy (4). Another neuromodulatory method, electroconvulsive therapy (ECT), failed to show a specific reduction in hallucination severity (7). In a similar vein, the effectiveness of repetitive transcranial magnetic stimulation (rTMS) is still highly controversial (8–10). Consequently, AVHs remain one of the central targets in treatment of schizophrenia, including experimental therapies [e.g., Ref. (11)].

Neurofeedback (NF) based on real-time functional magnetic resonance imaging (rt-fMRI) is a newly emerging technique with high potential for clinical applications in psychiatric disorders (12, 13). It allows subjects to voluntarily change the activity in a selected brain region [for a review, see Ref. (14, 15)] and elicit behavioral changes [for a review, see Ref. (16)]. In the first study applying fMRI-based NF in schizophrenia, nine patients learned to regulate their hemodynamic response in bilateral anterior insula (17). The training improved patients’ abilities to recognize disgust faces, but they were less accurate in recognition of happy faces. Although the study did not focus on clinical improvement, it showed patients’ ability to learn volitional brain control using rt-fMRI. A clinical improvement in positive and negative symptoms of schizophrenia was demonstrated in electroencephalography (EEG)-guided NF, with its effects evoking long-lasting results confirmed in a 2-year follow-up (18). fMRI-based NF has the potential for higher specificity and has been suggested as experimental treatment for AVH in addition to and in combination with its EEG-based counterpart (11).

To determine the underlying pathophysiology, neuroimaging studies on AVHs in schizophrenia have been carried out for over 25 years, demonstrating an involvement of a wide network of brain areas including secondary (and occasionally primary) sensory cortices, prefrontal and premotor cortex, anterior cingulate cortex (ACC), as well as subcortical and cerebellar regions [for a review, see Ref. (3, 19)]. Among them, the ACC has a key role in regulating emotions, goal-directed behaviors, attentional processes, response selection, online source monitoring, and cognitive control (20, 21). Moreover, the ACC is involved in differentiating between self- and not self-related stimuli (22), placing it as a good candidate to differentiate between inner speech and external stimulation. Furthermore, a recent meta-analysis on AVHs demonstrated decreased ACC activity in hallucinating as compared to non-hallucinating schizophrenia patients and healthy subjects (23). Although other brain regions, such as the superior temporal gyrus [STG (24, 25)] or the inferior frontal gyrus [Broca’s area (24)], may be equally important in psychopathology of AVH, we chose ACC as a target region for our study. First, the ACC has shown feasibility for NF studies in healthy subjects (26–31) and even in patients with schizophrenia (32, 33). Second, our research group has expertise in fMRI NF of this region, e.g., the successful upregulation of the rostral ACC was associated with increase in positive affect (27, 31) and improvement of emotional perception of voices (27).

Within the current study, we aimed to explore feasibility and the variability of effects of ACC regulation on AVHs. Therefore, three patients with schizophrenia, suffering from treatment-resistant AVHs, learned voluntary control over ACC activity using fMRI-based NF. We expected that successful upregulation will lead to a decrease in severity of AVHs, to improvement of psychopathology, particularly concerning positive symptoms, and to an increased valence of the perceived voices [in accordance with Ref. (27)].

## Materials and Methods

### Participants

Three right-handed patients with schizophrenia who suffered from different types of treatment-resistant AVHs participated as pilot subjects in the study. All three patients were diagnosed with schizophrenia according to the Structured Clinical Interview for DSM-IV-axis I disorders [SCID-I (34)]. They did not exhibit current psychiatric or neurological comorbidities. Severity of positive and negative symptomatology, as well as general psychopathology, was assessed by the Positive and Negative Syndrome Scale [PANSS (35)]. In order to assess the effect of NF training on AVHs and affect, directly before and after the measurement on each day, we examined severity of the AVHs using the Auditory Vocal Hallucination Rating Scale [AVHRS (36)], while current affective state was assessed with the PANAS (37). Additionally, patients were interviewed about the strategies used for regulation. After the measurement on each training day, they were asked to specify on a 10-point scale the degree of subjectively perceived control over their ACC activity (0 = no control at all and 10 = absolute control).

The patients were naïve to NF. Written informed consent was obtained prior to participation. The study protocol was approved by the Ethics Committee of the Medical Faculty of the RWTH Aachen University, Germany, in accordance to the Declaration of Helsinki.

#### Patient 1

##### Patient Demographics

The first patient was a 49-year-old woman. She had a polytechnic degree but was receiving early retirement pension. She had no partner.

##### Symptomatology

The patient was diagnosed with schizophrenia of the undifferentiated subtype. Schizophrenia was first diagnosed 15 years ago, and she was continuously hearing voices since that time. Periodically, she also experienced tactile as well as olfactory hallucinations. She currently showed a mild form of paranoia (e.g., people talking about her). Over the years, she progressively developed a negative symptomatology with blunted affect, poverty of speech, and slight difficulties with abstract thinking (see Table 1). At the time point of the study, patient 1 was medicated with clozapine (450 mg/day), aripiprazole (22.5 mg/day), and citalopram (30 mg/day) on a stable dose for 5 months.

**Table 1:** **Demographic and psychopathological information of the patients**.

	Gender	Age	Education (years)	Diagnosis(ICD-10)	Duration AVHs(years)	PANSS pos.	PANSS neg.	PANSS pp.	Medication
Patient 1	Female	49	18	F20.34	15	12	18	32	Yes
Patient 2	Female	33	18	F20.94	1.5	19	7	26	No
Patient 3	Male	24	10	F20.04	4	18	20	31	Yes

*PANSS pos, PANSS positive score; PANSS neg, PANSS negative score; PANSS pp, PANSS general psychopathology score*.

##### Characteristics of Auditory Hallucinations (According to AVHRS)

Patient 1 was continuously hearing several voices simultaneously, often more than 10 different ones speaking at once. The voices were mostly commenting her behavior, affronting her, or devaluing her, but there were also positive voices encouraging her. The patient perceived to have no control at all over the voices and felt moderately disturbed by them.

#### Patient 2

##### Patient Characteristics

The second patient was a 33-year-old married woman with a university degree in sport sciences. At the time of the study, she was an occupational therapist working with children.

##### Symptomatology

The patient was diagnosed with a schizophrenic disorder, not otherwise specified. She had been hearing voices for 1½ years. In addition to auditory hallucinations, she sometimes experienced olfactory hallucinations such as smelling smoke. Further, she reported a lifetime diagnosis of posttraumatic stress disorder (PTSD) and one episode of a major depression. At inclusion, she demonstrated symptoms neither of depression nor of PTSD. Positive symptomatology was moderate and negative symptoms were absent. The patient was not medicated at the time of the study (see Table 1).

##### Characteristics of Auditory Hallucinations (According to AVHRS)

At the moment of the study, the patient heard six voices that commented her behavior. She heard the voices at least every hour and reported to sometimes have control over them. She reported that at times, she was able to voluntarily induce pleasant voices by imagining activities she likes. Further, she described that she could sometimes stop the voices by “telling them insistently to shut up” or by concentrating on something else.

#### Patient 3

##### Patient Characteristics

The third patient was a young man of 24 years, with a high-school degree. He was doing part-time jobs once in a while and had no partner. He was smoking about one package of cigarettes per day for the last 5 years. On the third day of the NF training, the patient stopped the measurement during the third NF run because he suddenly became frightened in the MR scanner. He heard a voice telling him to stop. Therefore, on the third day, we obtained data from only two NF runs and no posttest.

##### Symptomatology

Patient 3 had a diagnosis of schizophrenia of the paranoid-hallucinatory subtype. After consuming amphetamines at a party 4 years before the study, he has continuously heard voices. He had five hospitalizations since then. During adolescence, he consumed a variety of drugs, but mainly cannabis and amphetamines. During acute phases of the illness, he experienced visual hallucinations and strong paranoia as well. At inclusion, he was hospitalized and exhibited moderate positive and negative symptomatology (see Table 1). He was medicated with clozapine (400 mg) for almost 2 years. The dose had not been changed over the last 6 months.

##### Characteristics of Auditory Hallucinations (According to AVHRS)

The patient heard five different voices that were threatening him. He perceived the voices as ghosts, which were talking about death and giving him orders. The patient felt very disturbed by the voices and indicated strong suffering. He reported to have no control at all over the voices.

### Experimental Stimuli and Task

**Figure 1:**
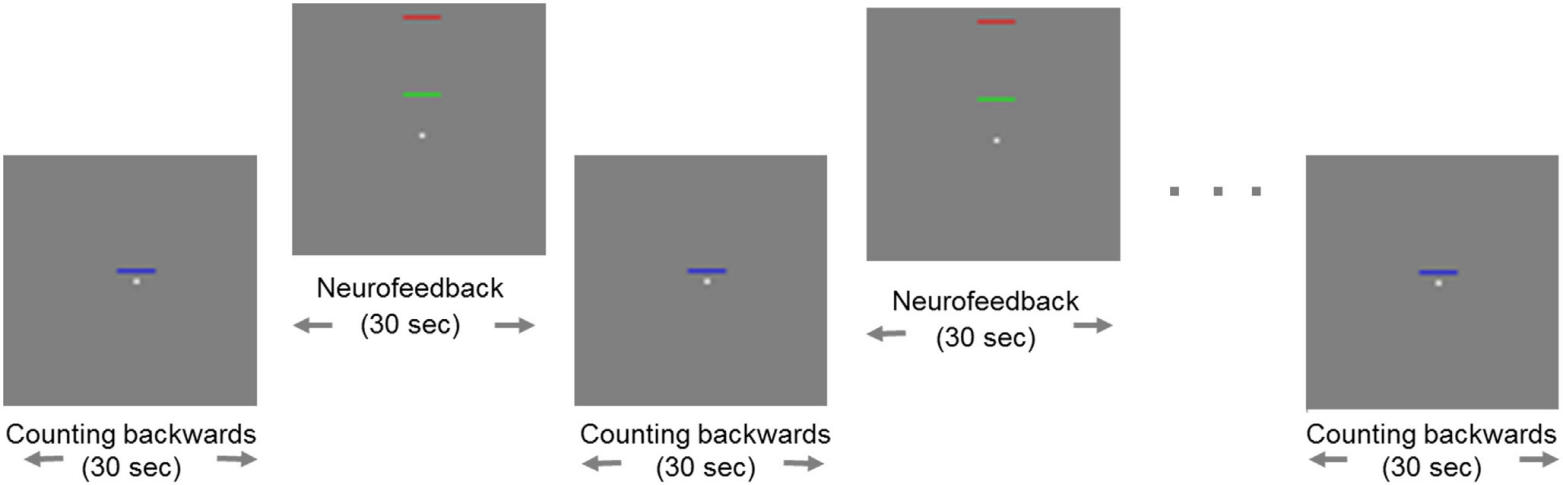
**Example of a neurofeedback run**. Blocks of regulation alternated with blocks of baseline (“counting”). During regulation blocks, the fixed red bar in the upper part of the screen served as a marker of the highest ACC activity as a target. The green moving bar indicated the current level of activity in the ACC, which should be moved toward the red bar. The baseline block was indicated by a static blue bar in the center of the screen.

All participants were trained to control activity in the ACC by means of rt-fMRI on three separate days within 1 week. On each day, they performed three NF training sessions, each consisting of eight regulation blocks and nine baseline blocks (30 s each; see exemplary run in Figure 1).

Change of activity in the ACC was indicated by a green bar moving up- or downwards, while a fixed blue bar indicated the baseline condition [see also Ref. (27)]. A fixed red bar in the regulation condition served as a regulation target. It indicated the upper limit of ACC upregulation. Subjects were instructed to raise the green bar in direction of the red bar using different mental strategies. During the baseline blocks, participants’ instruction was to count backwards from 100. The feedback was updated every repetition time (TR; 1 s).

Before the actual NF training, all subjects participated in a standardized training of mental strategies for ACC self-control. They received standardized instructions to recall positive emotional autobiographical memories, to imagine performing their hobby (such as engaging in sportive or musical exercise), or to concentrate on a specific perception (such as the temperature in one of their feet) in order to increase the activity in the region of interest (ROI). These strategies already proved effective in facilitating the NF training in previous studies [e.g., Ref. (32)]. The NF procedure was explained to subjects, including the delay of the NF signal for 3–5 s due to the hemodynamic response and data processing (<1 s).

Identical pre- and posttests assessed a possible transfer of the learned regulation. During these tests, subjects were asked to regulate their ACC activity with no feedback provided. The stimulus from the regulation training (i.e., the green bar) was presented during this transfer task in a fixed position. The baseline stimulus (blue bar) indicated the counting-backwards task. In the pre- and the posttest, there were four blocks of regulation and five blocks of baseline each. The posttest also included a cognitive visuospatial interference task, an adapted version of the Simon task, to test for generalization of the NF training to a behavioral task. However, single subject analysis of these cognitive interference data were not expected to yield results and were therefore not analyzed. The task for pre- and posttest was programed with Presentation software (Version 16.3).^1^

### Data Acquisition and Analyses

Functional magnetic resonance imaging was conducted using a 3-T whole body scanner (Magnetom TRIO, Siemens, Erlangen, Germany). We used a 12-channel array coil for the measurements. Sixteen transverse slices parallel to anterior commissure–posterior commissure (AC–PC) line were acquired with echo planar imaging (EPI) at a TR of 1 s (echo time TE = 28 ms; 64 × 64 matrix with 3 mm × 3 mm resolution; 3-mm slice thickness plus 0.75-mm gap). We obtained 520 volumes for each NF run (about 8.5 min) and 760 volumes for the pre- and posttest (12.5 min). A custom anatomical template mask of the ACC defined our ROI [for details, see Ref. (30)]. This ACC mask was taken as a part of the cingulate cortex as defined in the WFU Pickatlas Toolbox (38) and delineated by MNI coordinates anterior to *y* = 0 and superior to *z* = 0, yielding a volume of 15.1 ml.

The feedback signal was averaged across the ACC mask for each volume, calculated as percentage of signal change relative to the preceding baseline block and scaled that 1% represented the full regulation width. Online processing was conducted using a custom toolbox based on standard SPM procedure (39). In short, motion correction using spline interpolation with co-registration to the preselected template was implemented. A modified Kalman filter reduced outliers and high-frequency fluctuations. Low-frequency drifts were removed with an exponential moving average algorithm to improve the signal-to-noise ratio.

Offline data analysis was performed in SPM8 (FIL).^2^ The first 10 volumes of each run were excluded from the analyses accounting for T1 saturation effects. Data were realigned, normalized, and smoothed with an 8-mm Gaussian kernel. All different conditions were modeled in a block design applying a generic hemodynamic response function. Within a general linear model, T-maps for contrasts of interest (*regulation versus counting*, *posttest versus pretest*) were computed separately for each day and corrected for multiple comparisons across the volume using family-wise error (FWE) correction at a corrected threshold of *p* < 0.05 and an extended threshold of 15 voxels.

#### ROI Analyses

Next to whole-brain analyses, we performed ROI analyses for the different contrasts of interest, first, in the target ROI, i.e., the ACC. Second, activations within the reward system measured reinforcement by the NF. These ROI masks were generated from the WFU Pickatlas Toolbox (38). The mask for the ACC was as specified above, and the reward system contained the putamen, the caudate nucleus, and the globus pallidus. We determined the peaks within both ROIs on the F-maps (across all runs of all days). Activations in this peak voxel were then plotted over the different training days and for the transfer conditions.

## Results

### Behavioral Outcomes

#### Patient 1

##### Debriefing of Applied Strategies and Subjective Feeling of Control During NF

**Figure 2:**
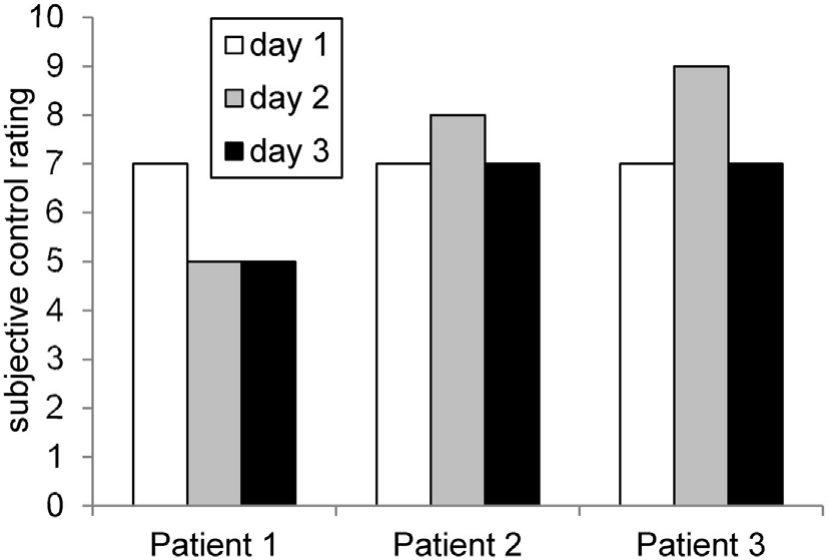
**Rating of subjective control over feedback signal across days**. The profiles matched roughly the achieved ACC regulation (compare Figure 6).

On the first day, patient 1 reported the strategy of imagining she was cycling. Additionally, she asked one of her favorite positive voices for help. On the subsequent days, she mainly used the strategy of concentrating on her positive voice and was not able to find an alternative strategy. Subjective feelings of control over ACC activity was high on the first day and decreased subsequently (see Figure 2).

##### Changes in AVHRS Scores

After the first training day, negative voices were perceived as less intense. She was better able to ignore them, felt less disturbed by them, and suffered less. After the subsequent two training days, she reported a decrease in disturbance by and suffering from voices. However, she felt more restricted in her concentration due to the voices (see Table 2).

**Table 2:** **AVHRS before and after neurofeedback training on days 1, 2, and 3 (improvements in bold)**.

Patient	Number of voices		Valence of voices^a^		Suffering from voices^b^		Disturbance by voices^b^		Control over voices^b^		Fear of voices^b^		Disturbed concentration by voices^b^	
**Day 1**														
	Pre	Post	Pre	Post	Pre	Post	Pre	Post	Pre	Post	Pre	Post	Pre	Post
1	5	5	**4**	**5**	**2**	**1**	**5**	**1**	5	5	0	0	1	2
2	**3**	**2**	**5**	**8**	**2**	**0**	**1**	**0**	4	0	0	0	0	0
3	1	1	**1**	**5**	**8**	**3**	**8**	**3**	2	2	0	0	**8**	**5**
**Day 2**														
	Pre	Post	Pre	Post	Pre	Post	Pre	Post	Pre	Post	Pre	Post	Pre	Post
1	5	5	5	5	**3**	**0**	**2**	**0**	**3**	**5**	0	0	2	3
2	0	0	0	0	0	0	0	0	0	0	0	0	0	0
3	**4**	**1**	5	5	**5**	**2**	**5**	**2**	2	2	0	0	**8**	**3**
**Day 3**														
	Pre	Post	Pre	Post	Pre	Post	Pre	Post	Pre	Post	Pre	Post	Pre	Post
1	3	5	5	5	**3**	**2**	**5**	**2**	5	5	2	2	**5**	**2**
2	**2**	**0**	**5**	**0**	0	0	0	0	2	0	0	0	0	0
3	1	2	5	5	2	2	2	2	2	2	1	1	**7**	**3**

*^a^Bipolar valence scale: 0 = very negative, 5 = neutral, and 10 = very positive*.

*^b^Unipolar 10-point scale: 0 = not at all and 10 = very much*.

##### Changes in PANAS Scores

**Figure 3:**
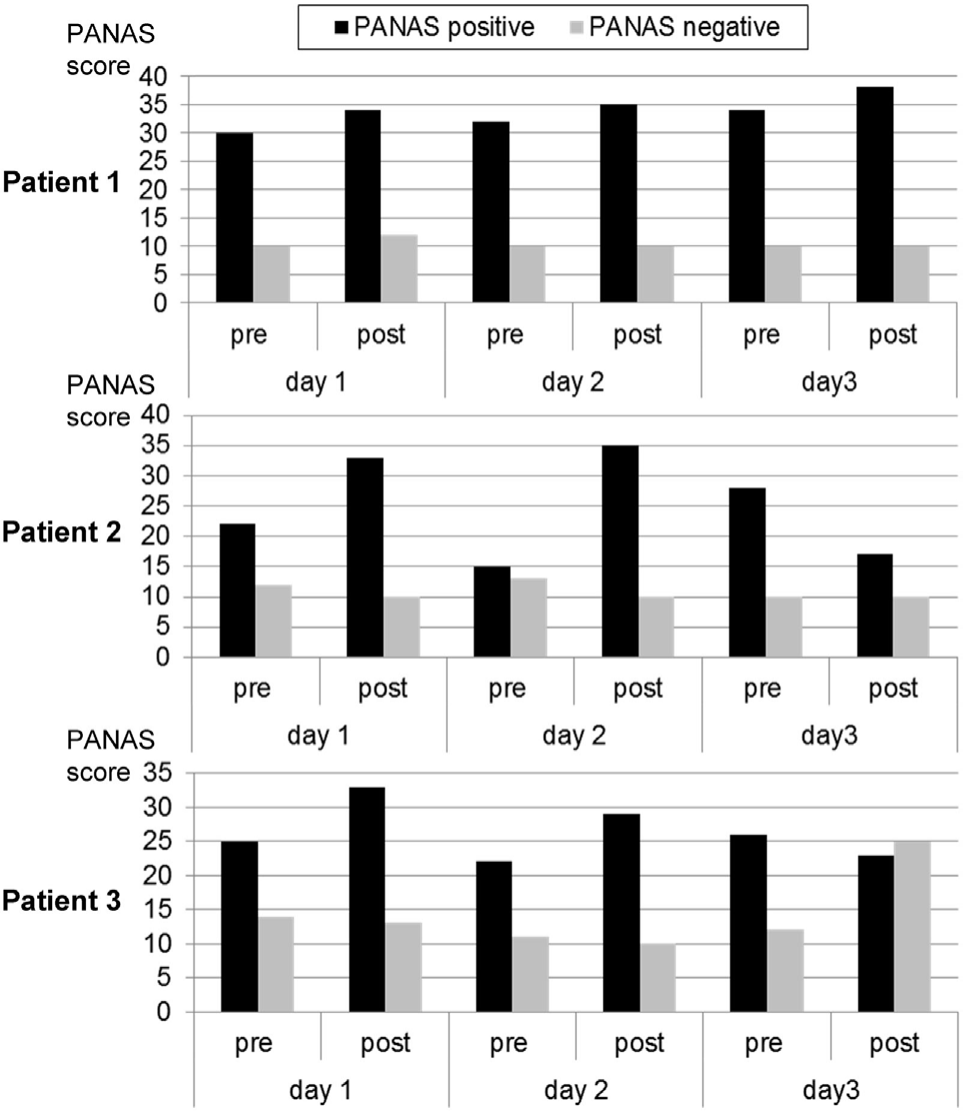
**Mood ratings according to the Positive and Negative Affect Scale (PANAS) before and after each neurofeedback training**. Patient 1 exhibited a trend for continuously improved positive affect. This was similar in the other two participants, except that the third day was associated with reduced positive or increased negative affect.

During each training day, positive mood indicators increased. Negative mood also increased on the first day and remained constant on days 2 and 3 (see Figure 3).

#### Patient 2

##### Debriefing of Applied Strategies and Subjective Feeling of Control during NF

On the first day, the patient imagined pleasant activities she did as a child, e.g., jumping into puddles and eating ice cream. On the subsequent days, she shifted her strategy and tried to concentrate on positive childish voices. On day 3, she imagined playing the guitar. Over all 3 days, she perceived to have control over her brain activity. The patient afterward stated that she applied similar strategies during daily life to induce pleasant voices as well. The score of subjective control over ACC activity increased slightly from day 1 to day 2 and decreased again on day 3 (see Figure 2).

##### Changes in AVHRS Scores

On the first day, she mainly reported a decrease in the perceived negativity of voices and small changes in suffering from voices and disturbance by voices. On the second day, there was no perceived change with respect to the voices, and on the third day, she reported an increase in perceived negativity of voices and a decrease of perceived control but also a decrease in the number of voices (see Table 2).

##### Changes in PANAS Scores

Over the first two training days, the patient reported an increase in positive mood and a reduction in negative mood. On the third day, she experienced a decrease in positive mood (see Figure 3).

#### Patient 3

##### Debriefing of Applied Strategies and Subjective Feeling of Control during NF

On the first training day, patient 3 reported imagining eating his favorite dish, thinking of holidays, meeting friends, or imagining his hand growing. On subsequent days, he repeated the same strategies and added imagining the exact appearance of his mother and friends as well as imagining the happiness when being discharged from the clinic. The subjective feeling of control over ACC activity increased from day 1 to day 2. On the third day, the control score decreased again (Figure 2).

##### Changes in AVHRS Scores

On the first and second days, he had the subjective feeling of a reduction of the presence and negativity of the voices during and shortly after the training. He also reported a decrease of suffering and disturbance by voices (by 5 points, respectively, on a 10-point scale) as well as a decrease in disturbances of concentration by voices. Additionally, he indicated a decrease in the number of voices heard (see Table 2 for exact scores).

##### Changes in PANAS Scores

On the first 2 days, the patient reported an increase in positive mood over the course of the training. Also, his negative mood decreased. After the break-up at the third day, he reported a decrease in positive mood and a strong increase in negative mood (see Figure 3).

### fMRI Results

#### Whole-Brain Analyses

**Figure 4:**
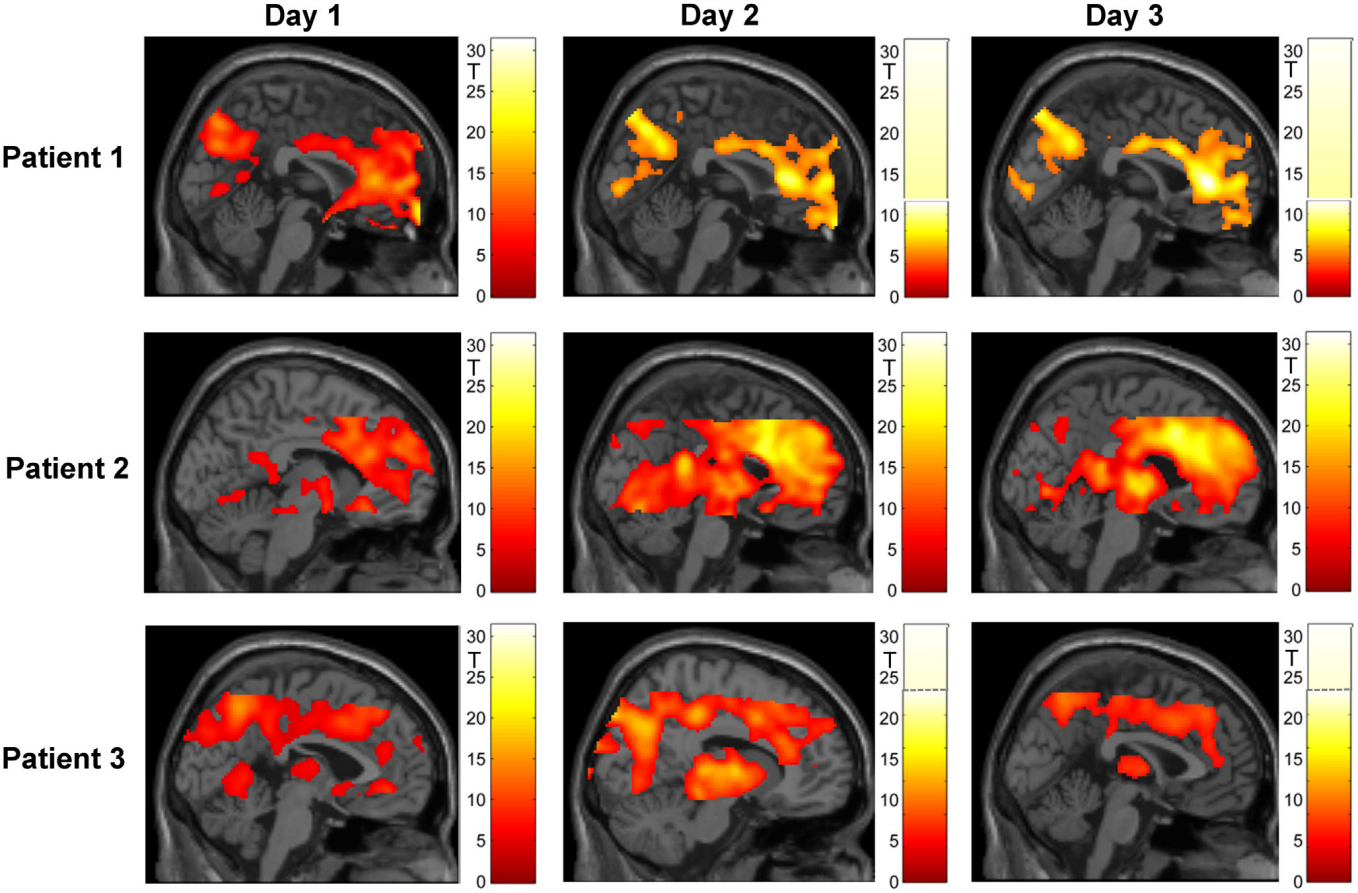
**Regulation during the neurofeedback runs as compared to baseline blocks of counting (*p* < 0.05, FWE corrected)**. The activation patterns did not reveal a clear increase in regulation, but the individual clusters appeared more focused on the ACC over the 3 days of neurofeedback training in patients 2 and 3. Please note that absolute *T* values differ across training days and across patients (adapted color codes). However, the identical thresholds make the cluster sizes directly comparable.

Figure 4 shows an overview of whole-brain activations on the three training days in the three subjects. During regulation as compared to the counting blocks, all patients yielded significant activation clusters within the ACC as well as the reward system, visual processing areas, the precuneus, motor areas, the amygdala, and the hippocampus.

##### ROI Analysis – ACC

###### Patient 1

**Figure 5:**
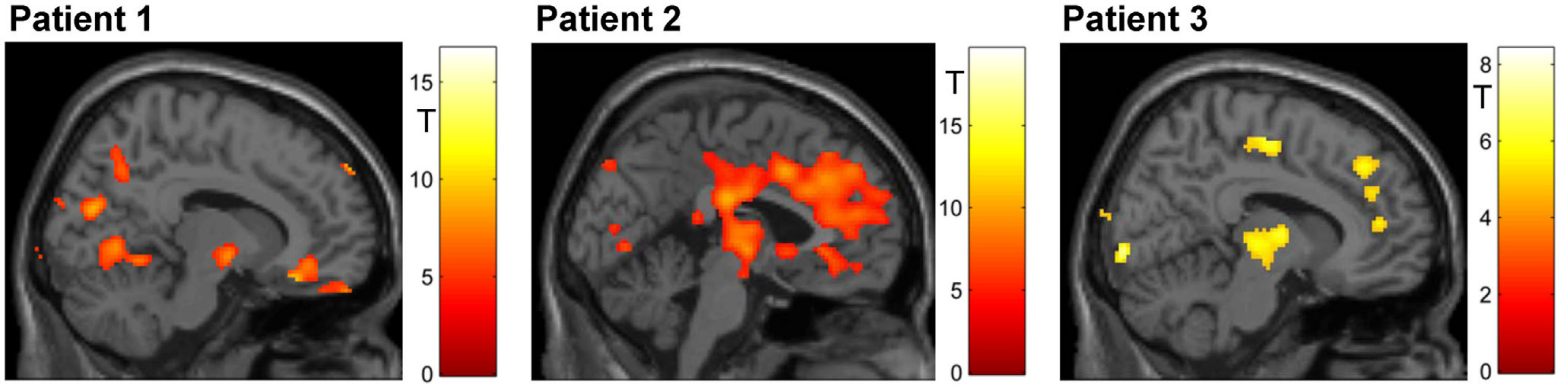
**Mapping of linear increase over days (*p* < 0.05, FWE corrected)**. The training led to increased activity in clusters encompassing midbrain structures (all patients) and – in patients 2 and 3 – in the ACC.

**Figure 6:**
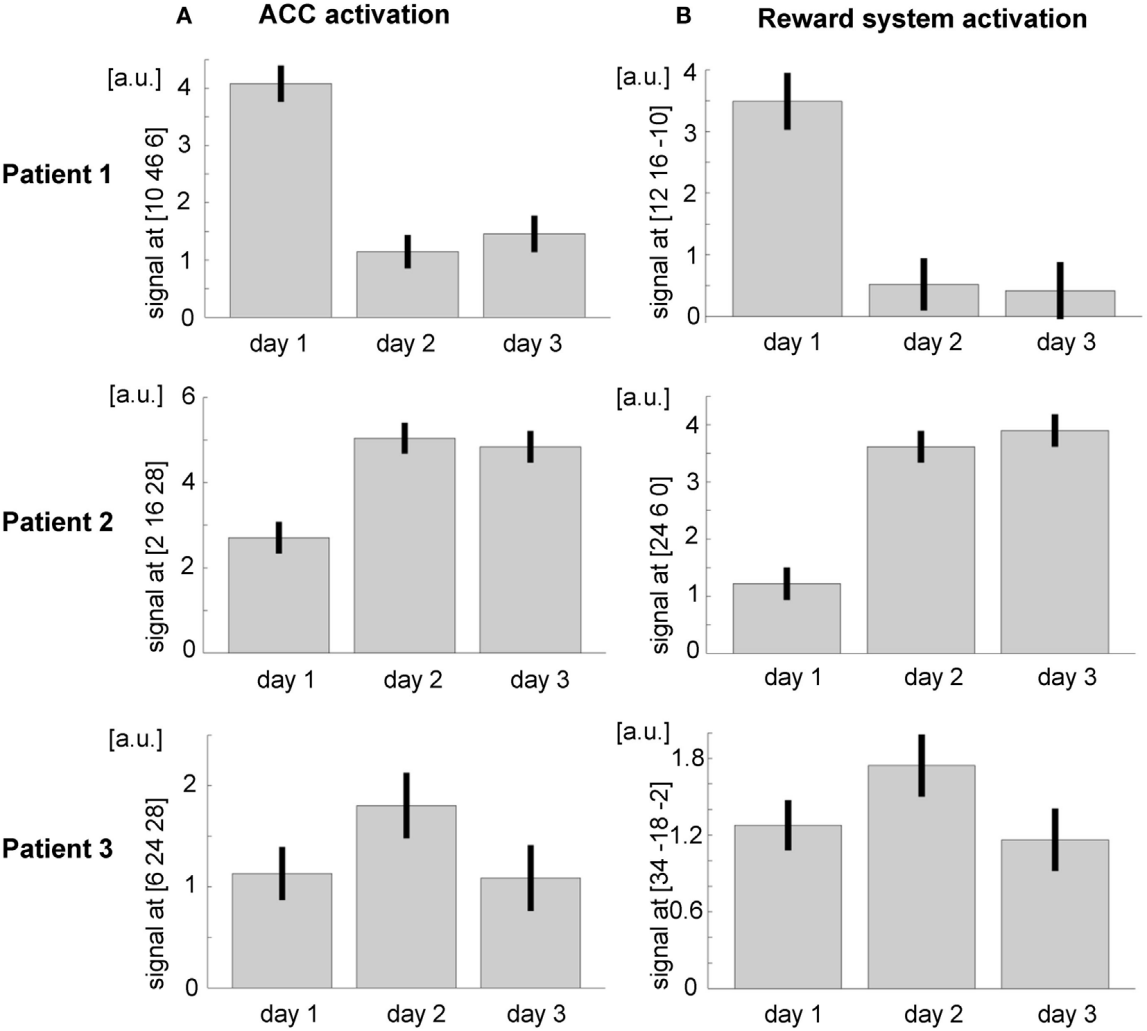
**Region of interest (ROI) analysis (A) in the ACC and (B) in the reward system revealed a general trend for increasing activity over the three training days**. However, the interindividual differences are remarkable; a.u., arbitrary units.

Within the ACC mask, significant activation emerged during the first day (*t*_peak_ = 20.92, *p*_FWE_ < 0.0001, MNI [10, 46, 6], 2474 voxels activated), during the second day (*t*_peak_ = 9.69, *p*_FWE_ < 0.0001, MNI [2, 32, 10], 1496 voxels activated), and during the third day (*t*_peak_ = 11.16, *p*_FWE_ < 0.0001, MNI [2, 34, 8], 1777 voxels activated). When directly comparing activation during self-regulation at the third and the first days, the patient did not show any significant activation at the ACC. At an exploratory uncorrected threshold of *p* < 0.001, one small cluster emerged in the subgenual ACC that was, however, not included in the ACC mask used in the training (MNI [10, 36, −10], *t*_peak_ = 6.88, *p*_uncorr._ < 0.001, 28 voxels activated; see Figure 5). The overall *F*-test revealed peak ACC activation across all runs of all three training days at an anterior part of the ACC (*F*_peak_ = 64.3, *p*_FWE_ < 0.0001; MNI coordinate = [10, 46, 6]). The ACC activation at this peak voxel over the 3 days indicated successful ACC regulation on the first day and then decreasing activation over the subsequent days (see Figure 6A).

Within the transfer task – comparing activation at posttest with that at the pretest – patient 1 did not show any ACC activation.

###### Patient 2

Also in patient 2, the ACC ROI showed significant activation during the first day (*t*_peak_ = 13.62, *p*_FWE_ < 0.0001, MNI [−6, 28, 24], 2156 voxels activated), during the second day (*t*_peak_ = 23.13, *p*_FWE_ < 0.0001, MNI [−2, 14, 28], 2770 voxels activated), and during the third day (*t*_peak_ = 22.01, *p*_FWE_ < 0.0001, MNI [−2, 28, 26], 2719 voxels activated). As already indicated by the *T* values, ACC activation during the third day was significantly stronger than during the first day (*t*_peak_ = 10.14, *p*_FWE_ < 0.0001, MNI [8, 12, −8], 1482 voxels activated; see Figure 5). An *F*-test across all training runs revealed peak ACC activation at a relatively posterior part of the ACC (*F*_peak_ = 133.54.3, *p*_FWE_ < 0.0001; MNI [2, 16, 28]). The bar plot evidences an increase of ACC activation within this peak voxel over the first two training days and constant regulation at the third training day (Figure 6A).

**Figure 7:**
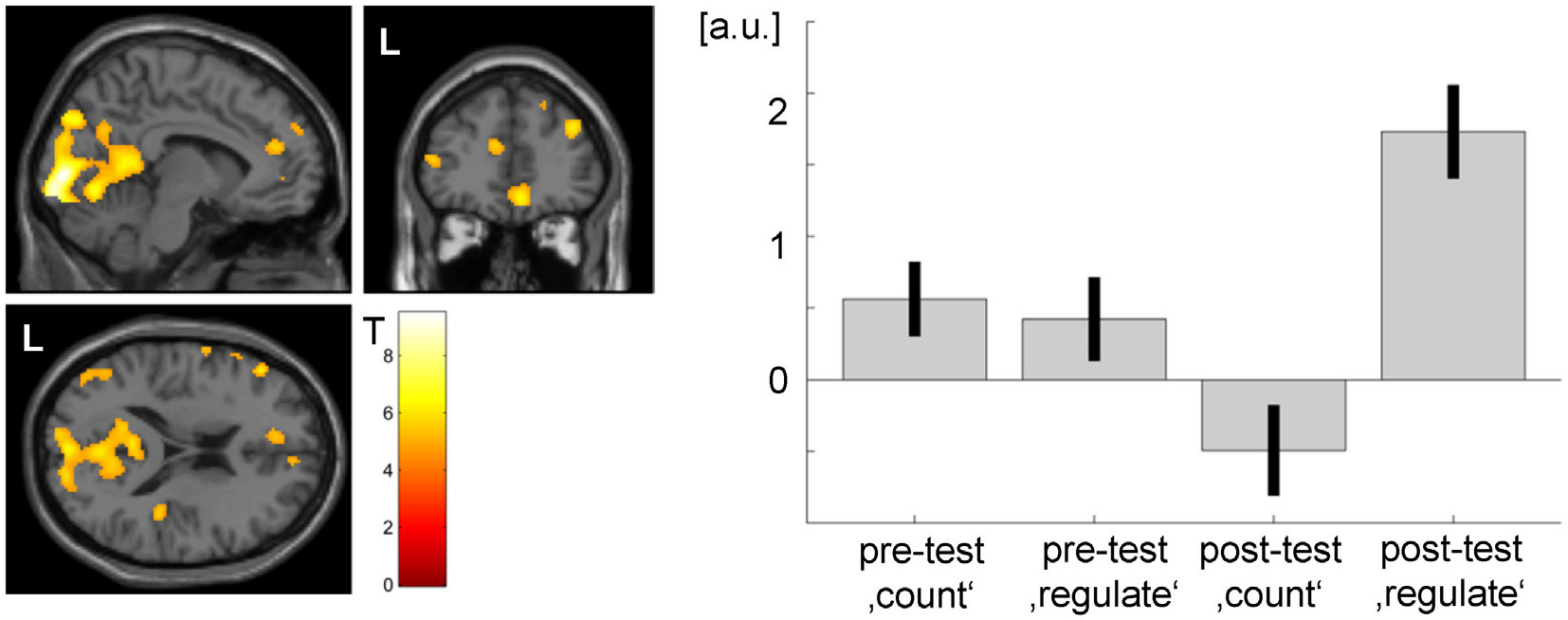
**Transfer effect in patient 2 (*p* < 0.05, FWE corrected)**. ACC regulation emerged also in the absence of feedback after the NF training as compared to before. However, the ACC activation cluster was noticeably smaller than during the neurofeedback training (see Figure 3) and did not yield significance in patient 1. The strong activation changes in the visual system may be related to attention shifts with respect to the visual task.

Comparing activation of the post- and the pretest within the transfer task, patient 2 showed significant ACC activation (see Figure 7). Bar plots indicate that during the pretest, there was no difference in peak ACC activation between regulation and baseline blocks. During the posttest, there was a significant increase in peak ACC activation during the regulation phases and a slight deactivation during the baseline period.

###### Patient 3

Within the ACC mask, T-maps confirmed significant activation during the first day (*t*_peak_ = 10.66, *p*_FWE_ < 0.0001, MNI [−4, 42, −8], 717 voxels activated), during the second day (MNI [10, 26, 26], *t*_peak_ = 9.79, *p*_FWE_ < 0.0001, 839 voxels activated), and during the two runs completed at the third day (MNI [8, 44, 10], *t*_peak_ = 6.4, *p*_FWE_ < 0.0001, 352 voxels activated). When directly comparing ACC activation between the first two runs of the third and the first day, there was an increase of ACC activation (*t*_peak_ = 5.64, *p*_FWE_ < 0.0001, MNI [−2, 36, 28], 67 voxels activated; see Figure 5). The *F*-test across all training runs revealed peak ACC activation at a relatively posterior part of the ACC (*F*_peak_ = 23.28.3, *p*_FWE_ < 0.0001; MNI [6, 24, 28]). Bar plots indicate that peak voxel activation increased from the first to the second day. On day 3, decrease may be observed (see Figure 6A).

The third patient finished the third training day earlier, and no data of the posttest are available; thus, we could not calculate the transfer effect for this patient.

##### ROI Analysis – Reward System

###### Patient 1

The overall *F*-test shows activation in four different clusters with peak activation at the MNI coordinate [12, 16, −10]. Inspecting activation within this peak voxel over the three training days indicated reward system activation on the first day and almost no activation during the second and the third days (see Figure 6B). When directly comparing activation of day 3 and day 1 within the ROI mask, significant activation could only be shown in a small cluster in the left putamen (MNI [−22, 14, −10], *t*_peak_ = 8.80, *p*_FWE_ < 0.0001, 68 voxels activated; see Figure 5B).

###### Patient 2

With respect to the reward system, the *F*-test shows activation in two clusters with the peak activation at the MNI coordinate [24, 6, 0]. Activation in this peak voxel across training days showed a very similar increase as the ACC. A direct comparison of ROI activation at day 3 and day 1 indicated significant bilateral activation (*t*_peak_ = 13.23, *p*_FWE_ < 0.0001, MNI [−32, 2, −6], 3331 voxels activated; see Figure 6B).

###### Patient 3

The *F*-test shows bilateral activation with peak activation at the MNI coordinate [34, −18, −2]. Bar plots showed similar bilateral activation increases from day 1 to day 2 and a drop in activation on day 3. Comparing activations of day 2 and day 1 with T-maps indicated a significant activation increase (MNI [28, −16, 4], *t*_peak_ = 6.66, *p*_FWE_ < 0.0001, 231 voxels activated; see Figure 6B).

## Discussion

We demonstrated the feasibility of fMRI-based NF training in patients with ongoing AVHs. After the NF training, the patients reported varied degree of subjective improvement concerning disturbance through and suffering from voices, as well as decrease in their number, intensity, and negativity. In combination with previous studies employing the same paradigm, the data suggest that patients with schizophrenia can learn localized control of ACC activity as well. For the patients suffering from treatment-resistant hallucinations for years despite intensive multimodal treatment, this new approach may change their understanding of AVHs, offering subjective experience of control over the voices. Nevertheless, even those three patients exhibited remarkable individual response patterns suggesting the need for a careful patient and paradigm selection for future clinical trials. More studies are necessary to assess the clinical relevance of fMRI NF, possibly in combination with other therapeutic techniques such as cognitive behavioral therapy.

The heterogeneity between the patients was also reflected in individually differing brain networks that were activated during NF. When evaluating mood changes across the NF training, the regulation training led to increased positive affect on the first and second days such as in a previous study with 1-day training (27). However, the participants did not profit from the prolonged three-session training. Indeed, in one subject, we even observed deterioration on the third day. Similarly, Surmeli et al. (18) demonstrated in their EEG-based NF study variability both in training duration and in clinical outcome. The authors postulate that every EEG-NF treatment protocol should be personalized to the specific patient and be regularly monitored and adjusted for an optimum treatment effect. The National Advisory Mental Health Council’s Workgroup (40) urged to search for personalized therapy, based on the knowledge about the individual that differentially predicts his or her response to treatment.

### Psychopathology and Neurofeedback

Personalization of treatment may be of particular interest considering the high interindividual variability between patients with schizophrenia that goes along with variability in individual activations patterns in the current study. In fact, the three patients measured in the current study showed high variability: next to differences in the exact diagnosis and duration of illness, also extrinsic factors, such as “secondary” negative symptoms, additional lifetime diagnosis of depression, and drug-related changes, may have caused further variability within the schizophrenia continuum. In accordance with the Report of the National Advisory Mental Health Council’s Workgroup (40), we describe below case-oriented results in hope that they provide a more stratified approach for linking psychopathology to treatment outcome and will complement standard clinical trials.

### Patient 1

Patient 1 showed successful regulation already in the first run of the first day but failed to increase the ACC activation over the course of training. The patient’s symptoms were concordant with a deficit form of schizophrenia, i.e., schizophrenia with primary and enduring negative symptoms (41, 42). This patient presented a long duration of illness with symptoms being stable over time. Additionally, she was the only patient treated with a highly D2-affine antipsychotic (aripiprazole). Negative symptoms and antipsychotic medication were found to be associated with reduced reward system sensitivity (43). Indeed, this patient exhibited much lower reward system activation during NF in comparison to the other two patients. In a previous animal study, reward system involvement was confirmed an obligatory requisite for learning to control BCIs (44). Although NF strongly relies on operant conditioning (16) and, therefore, on an adequate perception of rewarding events and appropriate reactions to rewards, the patient regulated ACC activity and demonstrated a consistent clinical improvement on all training days. Moreover, her subjective feeling of control over the ACC corresponded to actual ACC activity. Because negative symptoms in schizophrenia are often associated with attention deficits, it may also have been difficult for the patient to adopt, maintain, and shift the cognitive strategies as indicated by the feedback signals. This may explain why she did not show increasing ACC activation over the training and shifted from a successful regulation strategy on day 1 to less effective strategies on the subsequent days.

Auditory verbal hallucination symptoms showed a relatively close match with the training success on the 3 days [average blood-oxygen-level dependent (BOLD) effect in ACC]. Thus, the change in the AVH symptoms may also serve as a performance indicator and probably even stronger reward than the feedback from the bar display. Hence, future studies may probe to what extent strongly anhedonic patients profit from the instruction to consider an increase of the feedback signal and additionally explore specifically strategies to modify the depicted neuronal activity based on their AVHs.

### Patient 2

Patient 2 demonstrated both an increase of the ACC activation during NF from the first to the third day and a significant ACC upregulation in the transfer condition without feedback. These changes were accompanied by changes in the perception of her AVHs as well as changes in mood. While ACC activity increased on days 1 and 2 with concurrent improvements in the disturbance of AVHs, high activity on day 3 seemed to elucidate a trade-off between a decrease in the number of voices, an increase in the perceived negativity of the voices, and a decrease in mood. Insofar, the results from patient 2 do not show a clear correspondence between ACC activity and symptom experience on the third day but suggest that, at least for this patient, other brain circuitries may be required to yield constant effects on AVHs and mood. On the neuronal level, there was stronger concurrent activation of the anterior prefrontal cortex and left subgenual ACC at the first 2 days when compared to the third day. On day 3, in contrast, there was increased activation of the inferior frontal gyrus and insula. Thus, the improvement in AVH symptoms on the first days may have been due to an additional increase of prefrontal inhibition or prefrontally mediated focusing on the task (45–47). This patient might profit from feedback derived from several frontal brain areas or connectivity between the ACC and the anterior prefrontal cortex in order to achieve more stable effects of NF on symptomatology.

Importantly, after the NF training, this patient managed to upregulate her ACC even without receiving feedback. She was the only patient to show this transfer effect, which may be due to the fact that she already used similar strategies for the control of her voices before the study. If this transfer mechanism shows to be stable in the long term, it would allow voluntary control over AVHs also outside the scanner. Further, this finding would additionally strengthen long-term NF effects that may be due to neural plasticity (48). In a NF study with a small number of schizophrenia patients, Ruiz et al. (17) demonstrated this transfer effect on a trend level. In addition to increasing the number of patients, variability of patient characteristics should be considered in future studies.

### Patient 3

Patient 3 increased ACC regulation over the first two training days. These neuronal changes were accompanied by mood improvements as well as relevant changes in the perception of the AVHs. On day 3, he showed a decrease of ACC activation as well as a decrease in mood and no further perceived symptom reductions. Moreover, his subjective feeling of control over the ACC activity corresponded well with the actual ACC activity. Further, ACC activations were closely associated with the reported symptoms. However, this patient stopped the measurements on day 3 due to increased anxiety and warning of his voices. This leads to the question, if such “negative therapeutic response” (49) is an effect to consider in the NF treatment of AVHs. Indeed, many patients report AVHs as being at least in parts positive and helpful [e.g., Ref. (50)]. In combination with a growing fear to change positive aspects of the disorder, it needs to be considered whether 3 days of intensive rt-fMRI NF training within 1 week may be too exhausting for schizophrenia patients with complex symptoms. When inspecting the data, it seems that a 2-day training would have resulted in a comparable outcome. Conceivably, some patients might better profit from additional booster sessions of NF training some weeks later.

### Models of Auditory Verbal Hallucinations

Several theories have been put forward to explain the occurrence of AVHs and link them to observed neuronal changes. The feed-forward model being one of the most influential [for a review, see Ref. (3, 51)]. This neurocognitive model poses that AVHs occur due to a failure to recognize self-generated inner speech (51). The aberrant corollary discharge (or efferent copy) prevents comparing information about predicted action with received sensory input (52, 53). Consequently, self-generated speech is interpreted as externally generated (3, 54–57). Corresponding to the feed-forward model, patients with AVHs show systematic biases in motor tasks and pronounced deficits in cognitive tasks assessing self-recognition (58, 59). Indeed, connectivity was disrupted between temporal and cingulate cortices in schizophrenia patients with AVH only (60), and AVHs severity correlated to functional connectivity between the ACC and the STG (61). Contrary to patients with AVH, in healthy controls and in schizophrenia patients without AVH, the connectivity between left STG and the ACC (60) or the mPFC (62) was significantly greater for other – than for self-generated speech. Conceivably, in addition to structures, such as the STG, the ACC plays a central role in symptom generation and intensity modulation of AVHs.

However, the feed-forward model fails to explain phenomenological aspects of AVHs. For instance, most hallucinations are experienced as located in external space and take the form of another person’s voice, usually being experienced as “alien” (63). Cho and Wu (64, 65) pointed out that the internal articulation typically lacks properties associated with the experience of pitch and timbre distinct from one’s own voice, and they propose to replace the self-monitoring theory with the concept of auditory imagery as underlying phenomenon for AVH. AVH could also be linked to an externalizing bias in reality monitoring, which results in an impaired ability to distinguish between internally generated and externally generated percepts (66). Further, Waters et al. (67) conceptualizes AVHs as memory intrusions that are not recognized as such because they lack contextual cues. Another model attributes the emergence of AVHs to the highly increased salience of internal representations in schizophrenic patients that may be mediated by abnormal dopamine levels (68, 69). Hoffman et al. (70) proposes that prepsychotic social withdrawal prompts neuroplastic reorganization by the “social brain” to produce spurious social meaning *via* hallucinations of conversational speech. This diversity of models renders it difficult to select a fitting target region for voluntary brain regulation. Although most of the fMRI studies point to STG or auditory cortices as an evident target for downregulation (24, 25), it is not clear whether these areas can be downregulated to achieve a relevant physiological effect. Further, the downregulation of a sensory area would be equivalent of directing the attention away from a sensory channel, which patients already unsuccessfully tried before. It will be interested to target those regions in future fMRI NF studies to assess their feasibility and compare with the regulation of the ACC.

Previous NF studies demonstrated that the upregulation of a single area can elicit alterations of functional and effective connectivity (48, 71). Further studies may elucidate whether the ACC upregulation also induces changes of the network dynamics. In the long term, it may be even more effective if NF would target further areas and aim at a correlated regulation. First, this would allow addressing the entire dysfunctional neuronal ensemble that predisposes AVHs (3). And second, it would enable to adjust activity of the proposed salience network that may be of importance in the change of AVHs (72, 73).

### Other Neuromodulation Strategies

Despite the lack of hard evidence, EEG-based NF has been established in clinical domains such as attention deficit hyperactivity disorder (74) and epilepsy (75). For the application of EEG-based NF in schizophrenia, only case studies are available [e.g., Ref. (18)]. In general, these approaches are based on quantitative EEG, i.e., they attempt a “normalization” of oscillatory activity during resting state. This technique is widely available and relatively cost-effective. However, the training takes weeks to months [e.g., the average of 58.5 training sessions in Ref. (18)]. This is in an interesting difference to fMRI-based approaches. In our study and others [e.g., Ref. (76, 77)], successful control with fMRI-BCI tended to emerge extremely fast or even abrupt. It is unclear where this difference emerges from. Potentially, the precise localization supported by the modular organization of brain function enables a more direct control of the localized BOLD signal rather than EEG activity from distributed sources.

Alternatively, external neuromodulation strategies are available. In AVHs, particularly TMS has been applied. Typically, using repetitive stimulation to reduce local brain activity, rTMS studies targeted left temporoparietal cortex (10). Nevertheless, treatment success has been mixed, and improvements, such as localization based on fMRI activation maps, have been suggested (78). Moreover, TMS is limited to targets close to the surface; therefore, fMRI-based NF is ideally suited to base the target ROIs on such navigator [compare Ref. (79) for this strategy in depression]. The current study applied a predefined ROI of (parts of) the ACC. This region could not be addressed by TMS due to its anatomical localization. Indeed, such extended regions would not be assessable even to deep brain stimulation [DBS; see Ref. (80) for application in depression]. Finally, transcranial direct current stimulation (tDCS) is rather limited in targeting specific anatomical structures and to our knowledge has not been applied in AVHs yet. The combination of different neuromodulation strategies seems promising to optimize modulation strength, localization, and long-term effects, e.g., a combination of TMS and EEG-NF showed promising effects in autism (81). Such strategies could encompass TMS to localize the optimal modulation location, fMRI-NF to achieve rapidly a regulation strategy, and EEG-NF to maintain the learning effect over long term.

### Limitations

The most prominent limitation of the study is the small number of patients that does not allow for generalizing the findings to populations. However, the study is not aimed at demonstrating effectivity of the NF treatment or even making treatment suggestions. No systematic catamnesis was assessed because we did not expect persistent clinical affects from the one-time NF intervention. The study only provides evidence for the feasibility of ACC NF in patients with ongoing AVHs. The observed variability of clinical findings and the approaches to the feedback training offers information for the design of systematic studies, in particular clinical trials. It seems relevant to consider factors interfering with reward processing such as anhedonia and antipsychotic medication. Conceivably, the drug treatment alters the neuroplasticity or the ability to learn NF. Indeed, such a group of treated patients showed a different pattern in the learning of ACC regulation but quantitatively were as capable to learn ACC NF as matched controls (32). However, antipsychotics will be present in virtually all groups of schizophrenia patients with treatment-resistant AVHs. The effort for the patients to undergo the NF training should not be underestimated as well as the mixed motivation of the patients, since there are often also positive aspects of the hallucination, which the patients may fear losing after the training. Particularly, we cannot exclude the non-specific influence of undergoing the procedure, since there was no control patient group (e.g., who learned the control of a different brain region).

Further, no formal long-term follow-up was conducted with the patients, which is underrepresented in fMRI-BCI research in general (17, 71). In informal reassessments during clinical visits, none of the patients reported adverse effects and two of the patients claimed to have different strategies in dealing with their AVHs up to few weeks after the training. However, in contacts more than a month after the study, none of the patient had the impression that there was any influence on their symptoms. Individual variability and fluctuation in the disease course may override the – so far rather small – effects of the NF training. This may change with better targeted NF protocols. However, based on the clinical impression, we would suggest that at least monthly reminder session would be advisable for clinical trials.

Finally, other neural networks may serve as target for the NF training. The current pilot study did not compare the regulation of the ACC with other regions. Thus, unspecific processes may have engaged the ACC during the regulation attempts and contributed to the observed activation pattern. In particular, performance monitoring and related psychological functions may yield baseline activation during the regulation blocks. Nevertheless, NF is thought to – at least – increase these activations [e.g. Ref. (27)]. Further, experimental trials or case studies may be directed to alternative regulation targets in AVH. In particular, the auditory cortex may be an important target for downregulation [see Ref. (81)], but so far, there is little experience in self-regulation of these areas with fMRI-NF [see review in Ref. (82)].

## Conclusion

We explored the application of fMRI-based NF in therapy-resistant AVHs. Three patients suffering from AVHs due to schizophrenia trained the self-regulation of ACC activity. In general, they achieved control of areas encompassing the ACC, and in one patient, the transfer of regulation to a condition without NF could be shown. Further regulation success was associated with changes in symptom scores. However, variability of disease presentation and antipsychotic medication was reflected in the individual repose patterns. These data should inform future NF studies in AVHs; among others, such trials need to take into account individual differences in reward processing, fatigue, and motivation.

## Author Contributions

Idea and preparation: MD, KAM, YK, MZ, SS, and KM; clinical examination: MD, EA, and AG; data evaluation and interpretation: MD, KAM, PS, SB, and KM; manuscript writing: MD, KAM, SB, PS, and KM; and manuscript editing: all.

## Conflict of Interest Statement

The authors declare that the research was conducted in the absence of any commercial or financial relationships that could be construed as a potential conflict of interest.

## Footnotes

^1^www.neurobs.com

^2^http://www.fil.ion.ucl.ac.uk/spm/

## Abbreviations

ACC, anterior cingulate cortex; AC–PC, anterior commissure–posterior commissure; AVHRS, Auditory Vocal Hallucination Rating Scale; AVHs, auditory verbal hallucinations; BOLD, blood-oxygen-level dependent; DBS, deep brain stimulation; DSM-IV, diagnostic and statistical manual of mental disorders, 4th edition; ECT, electroconvulsive therapy; EEG, electroencephalography; EPI, echo planar imaging; FEW, family-wise error; MNI, Montreal Neurological Institute; NF, neurofeedback; PANAS, Positive and Negative Affect Schedule; PANSS, Positive and Negative Syndrome Scale; PTSD, posttraumatic stress disorder; ROI, region of interest; rt-fMRI, real-time functional magnetic resonance imaging; rTMS, repetitive transcranial magnetic stimulation; SCID-I, structured clinical interview-axis I; SPM, statistical parametric mapping; STG, superior temporal gyrus; tDCS, transcranial direct current stimulation; TR, repetition time.
